# Challenges in the diagnosis of Boerhaave syndrome: A case report

**DOI:** 10.1097/MD.0000000000018765

**Published:** 2020-01-10

**Authors:** Ching-Hsuane Tzeng, Wei-Kung Chen, Huei-Chun Lu, Hsin-Hung Chen, Kuan-I Lee, Yung-Shun Wu, Feng-You Lee

**Affiliations:** a Department of Emergency Medicine, Taichung Tzu Chi Hospital, Buddhist Tzu Chi Medical Foundation; b Department of Emergency Medicine, Trauma and Emergency Center, China Medical University Hospital, Taichung; c Department of Emergency Medicine, School of Medicine, Tzu Chi University; d Department of Emergency Medicine, Mennonite Christian Hospital, Hualien, Taiwan (R.O.C.).

**Keywords:** acute chest pain, Boerhaave syndrome, esophageal perforation, pneumomediastinum, pneumothorax, subcutaneous emphysema

## Abstract

**Rationale:**

Acute chest pain remains one of the most challenging complaints of patients presenting to emergency departments (EDs). The diverse etiologies of chest pain frequently lead to diagnostic and therapeutic challenges. Esophageal perforation is a rare but potentially life-threatening disease. It results in delayed diagnosis and an estimated mortality risk of 20% to 40%. Prompt diagnosis and immediate therapeutic interventions are key factors for a good prognosis.

**Patient concerns:**

Case 1 involved a 66-year-old man who presented to the ED with acute chest pain radiating to the back and hematemesis. Emergent contrast thoracic computerized tomography (CT) indicated the presence of a massive pneumothorax with pleural effusion. The continuous drainage of a dark-red bloody fluid following emergent thoracic intubation led to the discovery that the patient had experienced severe vomiting after whiskey consumption before admission to the hospital. Re-evaluation of the CT indicated spontaneous pneumomediastinum, whereas barium esophagography confirmed the presence of an esophageal perforation. Case 2 involved an 18-year-old Vietnamese man admitted to our ED with acute chest pain and swelling of the neck after vomiting due to beer consumption. A chest x-ray indicated diffuse subcutaneous emphysema of the neck and upper thorax. Contrast CT indicated pneumomediastinum with extensive emphysema and air in the paraspinal region and spinal canal.

**Diagnoses:**

Both of the 2 cases were diagnosed as spontaneous perforation of the esophagus (Boerhaave syndrome [BS]).

**Interventions:**

Case 1 received surgical interventions, whereas case 2 decided not to avail our medical services.

**Outcomes:**

Case 1 was discharged after a good recovery. Case 2 lost to follow-up.

**Lessons:**

We recommend all physicians in the ED to raise their index of suspicion for BS when dealing with patients having acute chest pain, dyspnea, confirmed pneumothorax, or newly-developed pleural effusion.

## 1 Introduction

Acute chest pain remains one of the most common and challenging complaints of patients presenting to emergency departments (EDs). In the United States, the number of ED visits for chest pain is approximately 8 million, which accounts for 5% of all ED visits per year.^[1–4]^ The diverse etiologies of acute chest pain range from acute coronary syndrome to various mimics, which frequently lead to challenges in diagnosis and therapy.^[3,5]^ Esophageal perforation is a rare but potentially life-threatening disease, which is easily missed during diagnosis.^[5]^ Full-thickness rupture of the esophagus results in the extravasation of gastric juice and oral secretions, which causes peri-esophageal space contamination, pleural space inflammation, and systemic sepsis. Esophageal perforation has an estimated mortality risk of 20% to 40%.^[6–8]^ Therefore, prompt diagnosis and appropriate immediate therapeutic interventions are key for a good prognosis.^[7]^

### 1.1 Case 1

A 66-year-old man presented to our ED complaining of acute chest pain radiating to the back accompanied with hematemesis. He had a past medical history of left-side breast cancer and had undergone total mastectomy with a sentinel lymph node biopsy 2 years ago. The patient was currently under oral Tamoxifen therapy.

**Figure 1:**
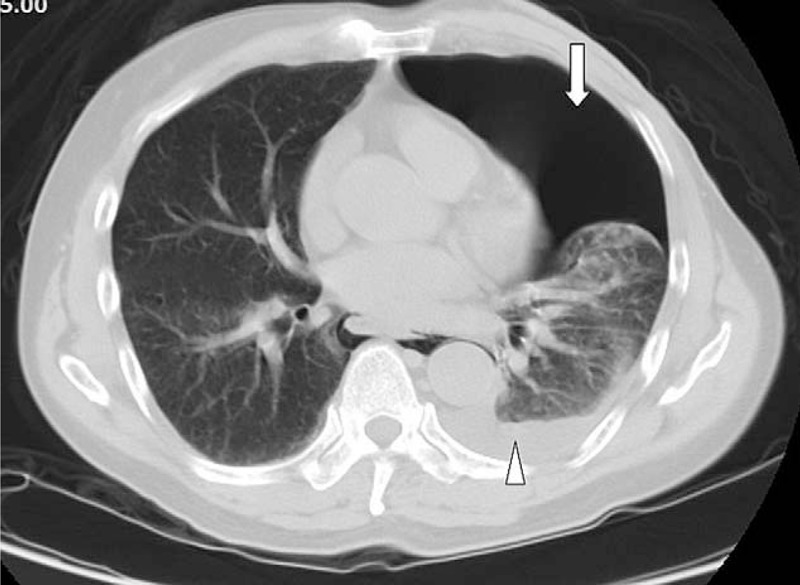
Emergent contrast thoracic computerized tomography (CT) demonstrating massive pneumothorax on the left side (arrow) with collapsed lung and mild pleural effusion (arrowhead).

**Figure 2:**
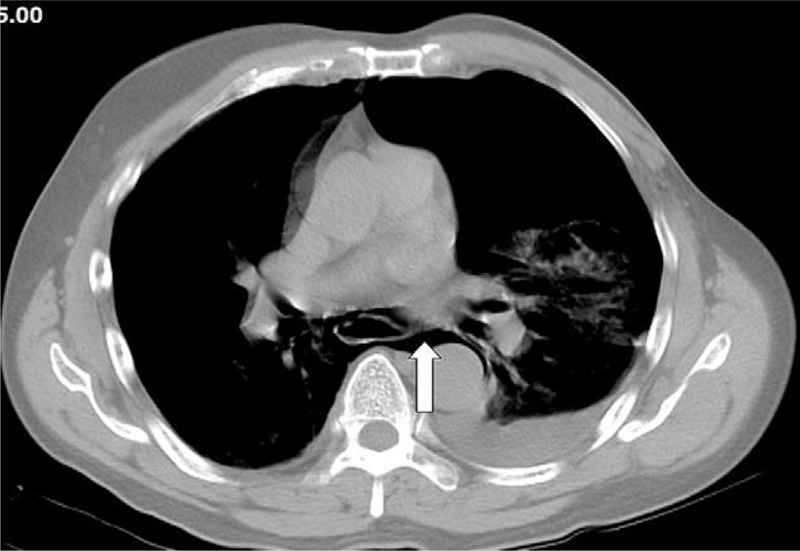
Contrast thoracic CT demonstrating massive extra-luminal air encompassing the peri-esophageal spaces (pneumomediastinum; arrow). CT = computerized tomography.

**Figure 3:**
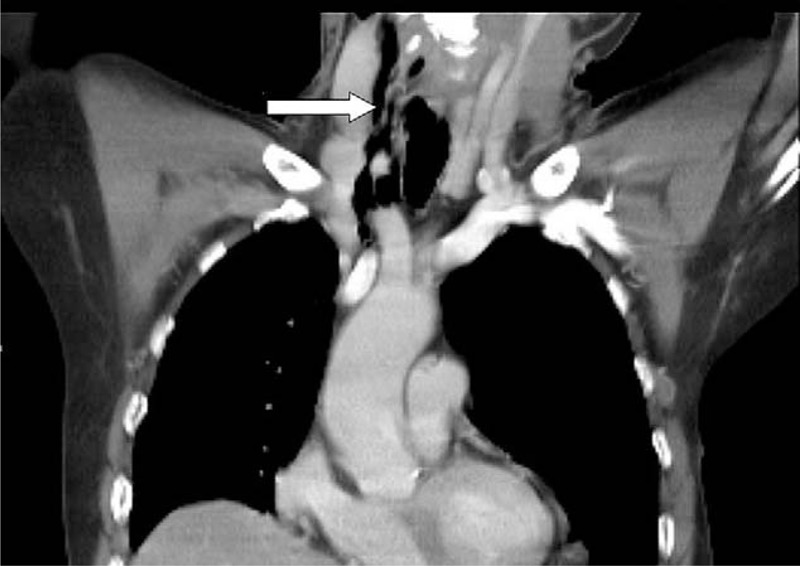
Coronal view of thoracic-CT revealed pneumomediastinum extending upward into the right lower neck region (arrow). CT = computerized tomography.

**Figure 4:**
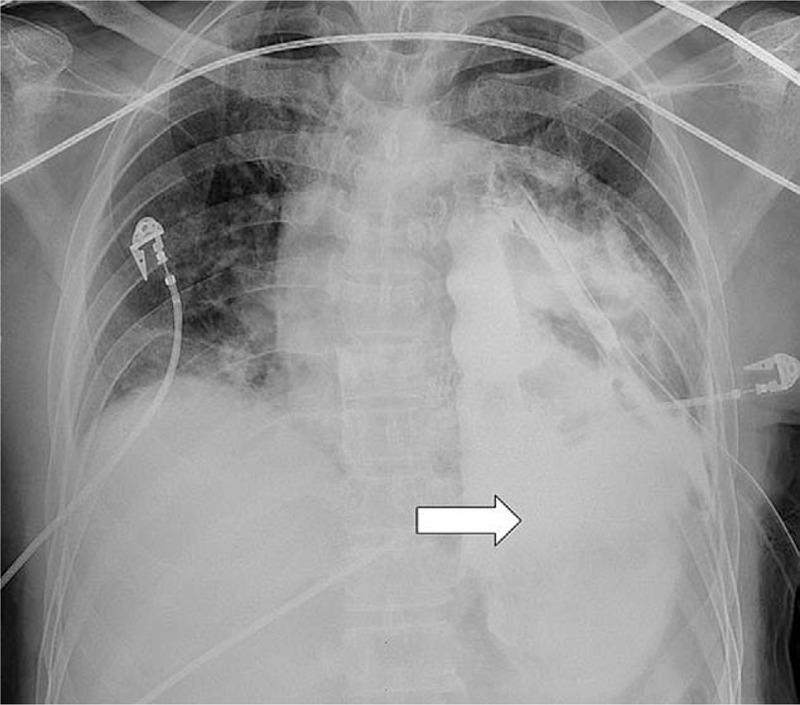
Barium esophagography exhibiting the leakage of the oral contrast in the left hemi-thorax cavity (arrow), which indicates esophageal perforation.

Upon arrival at the ED, the patient appeared restless and acutely ill. The vital signs were measured and appeared to be normal. Physical examination revealed a coarse and diminished breathing sound in the chest on the left side. No other remarkable findings were observed. Initial electrocardiogram examination indicated a normal sinus rhythm. Laboratory tests indicated only a marginal elevation in the white blood cell counts. Based on the suspicion of a ruptured aortic dissection, emergent contrast thoracic computerized tomography (CT) was performed. A massive pneumothorax with mild pleural effusion was observed on the left side (Fig. 1). Thus, a diagnosis of spontaneous pneumohydrothorax was reached and emergent thoracic intubation with drainage was performed. However, continuous dark-red bloody drainage from the tube was observed. Detailed information obtained from the family revealed that the patient experienced severe vomiting after consuming an excessive amount of whiskey several hours before admission to the hospital. The CT scan was carefully reviewed and pneumomediastinum (Fig. 2) extending upward into the right lower neck (Fig. 3) and downward into the esophagogastric junction region was identified. Barium esophagography demonstrated contrast leakage into the left hemi-thorax cavity (Fig. 4), which indicated esophageal perforation. The patient received surgical interventions including exploratory thoracotomy with the repair of the distal posterolateral esophageal laceration and feeding jejunostomy. The surgical interventions were accompanied with adequate nutrition support, pain management, and broad-spectrum antibiotic therapy. Esophagography performed 1 week after the surgical procedure revealed no further contrast leakage from the anastomosis site. The thoracostomy drainage tube was removed, and the patient was discharged with good recovery and in a stable condition.

### 1.2 Case 2

**Figure 5:**
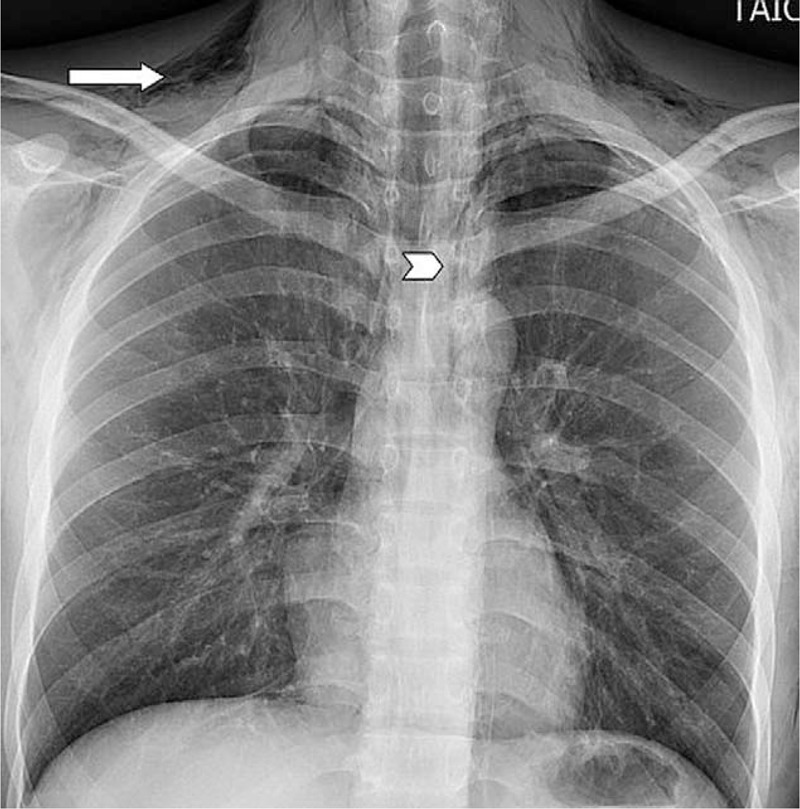
Chest x-ray revealing diffuse subcutaneous emphysema in the soft-tissue layers of the neck and upper thorax (arrow) and the linear distribution of air along the mediastinum (arrowhead).

**Figure 6:**
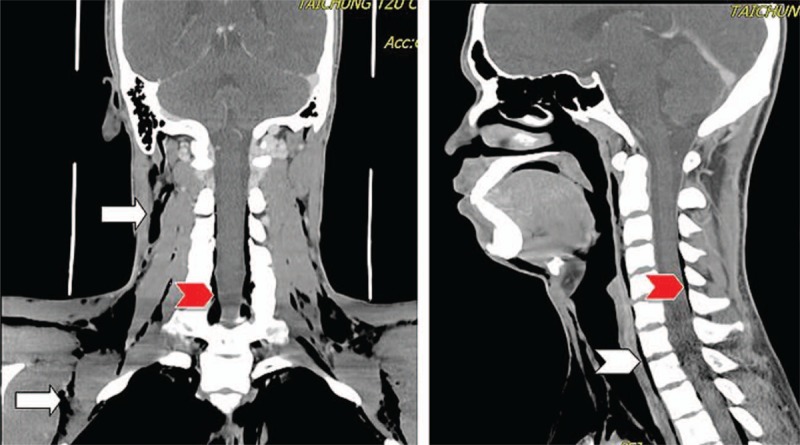
Contrast neck and thoracic CT demonstrating pneumomediastinum with extensive emphysema in the neck and chest wall (arrows). Free air was present in the paraspinal region (white arrowhead) and epidural space of the spinal canal (red arrowheads), which is termed as “pneumorrhachis.” CT = computerized tomography.

An 18-year-old Vietnamese man presented to our ED complaining of acute chest pain and swelling in the neck after several episodes of vomiting following beer consumption. The initial vital signs were normal except for a pulse rate of 125 beats/min. Physical examination revealed bilateral neck crepitus with tenderness. A chest x-ray indicated diffuse subcutaneous emphysema in the soft-tissue layers of the neck and upper thorax (Fig. 5). Emergent contrast CT demonstrated pneumomediastinum with extensive emphysema in the neck (bilateral), chest wall, paraspinal region, and spinal canal (Fig. 6). The patient was diagnosed with Boerhaave syndrome (BS) and immediate surgical or intensive medical treatment was recommended. However, the patient did not avail the consultation and treatment services provided by the hospital.

## 2 Discussion

Esophageal perforation is a rare condition; however, it has been recognized as the most sinister trauma of the gastrointestinal tract. The degree of severity ranges from a small leakage of air into the mediastinum to a more wide-spreading destruction with massive air and fluid breaching and draining to the pleural cavity, which leads to an overwhelming sepsis, shock, and death.^[8,9]^ BS (spontaneous barogenic perforation of the esophagus), was first reported by Dr. Herman Boerhaave in 1724.^[10]^ Differing from the pathologic rupture caused by malignant or inflammatory esophageal diseases, BS indicates the perforation of a previously healthy esophagus which is most frequently affected by forceful vomiting or retching after the consumption of large amounts of alcohol or food.^[10,11]^ The typical symptoms of BS include chest pain followed by vomiting or retching, and subcutaneous emphysema (Mackler triad); however, these symptoms generally occur in only 14% of all patients with BS.^[11–13]^ Therefore, the non-specific symptoms presented could easily confuse the emergency physician and lead to a missed or delayed diagnosis, which can result in a disastrous outcome. The mortality rate of BS has been reported to be 25% when appropriate intervention is activated within the first 24 hours of admission. The mortality rate sharply increases to almost 100% when precise diagnosis and interventions are delayed over a period of 48 hours.^[11,14]^ Surgery with primary esophageal repair and debridement remains the most effective method for treating BS. Other treatment options include endoscopic stenting/clipping, adequate pleural drainage, broad-spectrum antibiotics as well as sufficient nutrition support.^[10–12]^ Prompt diagnosis is considered a major determinant of good prognosis.^[6]^

In the first case presented in this report, spontaneous hemopneumothorax was first diagnosed immediately after a quick CT scan reading and emergent thoracostomy tube drainage was promptly performed. The development of this condition can result from thoracic malignancies, idiopathic pneumothorax with torn vascular adhesion band, and a variety of infectious events.^[15,16]^ However, we disregarded the potentially life-threatening cause for the disease at the time until a continuous drainage of dark-red bloody material was observed and additional information regarding a history of vomiting after alcohol consumption was obtained from the family. These factors raised our suspicions about a possible diagnosis of BS. Fortunately, the patient received timely thoracotomy with repairment of the esophageal rupture and debridement within the first 12 hours. A longitudinal laceration was documented over the left posterior lateral part in the lower third of the esophagus, which was in agreement with the most frequent site of perforation reported.^[10]^ The patient recovered well and was successfully discharged without complication. We recommend that emergency physicians should remember that BS could mimic spontaneous or even tension pneumohydrothorax.^[11]^ Inquiries about any precipitating factors, careful evaluations for signs of subcutaneous emphysema or sepsis, and arrangement of appropriate diagnostic tools are warranted for maximizing the chances of survival of patients with BS.^[8,10,11,17,18]^

Based on the experience from case 1, we could diagnose the highly suspicious case of BS in the second patient who presented with the typical Mackler triad and other promoting factors, including severe vomiting after beer consumption. As expected, the chest radiograph and focused CT scan displayed diffuse subcutaneous emphysema, pneumomediastinum, and notably, pneumorrhachis, which refers to the existence of air in the spinal canal or intervertebral foramens.^[19]^ Unfortunately, the patient refused any further treatment and was lost to follow up.

## 3 Conclusion

We recommend that all physicians at the ED should consider BS in the differential diagnosis when dealing with patients with acute chest pain, dyspnea, confirmed pneumothorax, or newly-developed pleural effusion. A high index of suspicion, thorough collection of the medical history, focused physical examination, suitable diagnostic tools, and the implementation of individualized treatment strategies are key factors for achieving a satisfactory outcome.

## Author contributions

**Conceptualization:** Huei-Chun Lu, Hsin-Hung Chen, Feng You Lee.

**Supervision:** Wei-Kung Chen, Kuan-I Lee.

**Writing – original draft:** Ching-Hsuane Tzeng.

**Writing – review & editing:** Yung-Shun Wu, Feng You Lee.

Feng You Lee orcid: 0000-0002-5824-1574.
